# Tunable magnetism in metal adsorbed fluorinated nanoporous graphene

**DOI:** 10.1038/srep31841

**Published:** 2016-08-24

**Authors:** Pankaj Kumar, Vinit Sharma, Fernando A. Reboredo, Li-Ming Yang, Raghani Pushpa

**Affiliations:** 1 Department of Physics, Boise State University, Boise, ID 83725, USA; 2 Materials Science and Technology Division, Oak Ridge National Laboratory, Oak Ridge, Tennessee 37831, USA; 3 School of Chemistry and Chemical Engineering, Huazhong University of Science and Technology, Wuhan 430074, China

## Abstract

Developing nanostructures with tunable magnetic states is crucial for designing novel data storage and quantum information devices. Using density functional theory, we investigate the thermodynamic stability and magnetic properties of tungsten adsorbed tri-vacancy fluorinated (TVF) graphene. We demonstrate a strong structure-property relationship and its response to external stimuli via defect engineering in graphene-based materials. Complex interplay between defect states and the chemisorbed atom results in a large magnetic moment of 7 μ_B_ along with high in-plane magneto-crystalline anisotropy energy (MAE) of 17 meV. Under the influence of electric field, spin crossover effect accompanied by a change in the MAE is observed. The ascribed change in spin-configuration is caused by the modification of exchange coupling between defect states and a change in the occupation of *d*-orbitals of the metal complex. Our predictions open a promising way towards controlling the magnetic properties in graphene based spintronic and non-volatile memory devices.

Understanding mechanisms that control magnetism at the nanoscale is crucial for designing novel spintronic and data storage devices. However, understanding these mechanisms remains an open challenge in material science, with a tremendous impact on today’s information technology^1^,^2^,^3^,^4^,^5^,^6^,^7^,^8^,^9^,^10^,^11^. Nanoscale magnetism is a complex interplay between magnetic anisotropy, exchange interactions, and quantum tunneling of magnetization, which in turn depend on the size, geometry, and composition of a nanostructure. One of the most promising methods to control the magnetic properties such as magneto-crystalline anisotropy energy (MAE) and magnetic moments of nanostructures is through an external electric field (e-field)^12^,^13^,^14^,^15^,^16^.

Graphene – a single sheet of carbon atoms has unique mechanical, electronic, and optical properties^17^,^18^, such as high mechanical strength, electronic mobility at room temperature^17^,^19^, long spin-relaxation lengths^20^,^21^, and most importantly the senstivity of its electronic properties toward an applied electric field^22^. These properties make graphene and its derivatives a potential candidate for spintronic and data storage devices. In graphene, carbon (C) atoms are *sp*^2^ hybridized in honeycomb geometry and exhibit a large surface area. The outer low-energy *p*_z_ orbitals of C atoms can hybridize with the defect states modulating its electronic and magnetic properties^22^,^23^. While pristine graphene is nonmagnetic, the presence of certain types of defects can induce magnetism by breaking the balance between two sub-lattices and creating unpaired spins^24^,^25^,^26^. Defects such as vacancies^27^,^28^,^29^,^30^,^31^,^32^ and light adatoms like H, F and transition metal (TM) atoms have been found to induce magnetism in graphene^33^,^34^,^35^,^36^. However, the presence of thermal fluctuations can spin flip the states in spin-electronic devices, which can limit the practical applications of these materials. Therefore, a large MAE is required to preserve the magnetism in two-dimensional (2D) systems.

In general, MAE is an interplay among spin-orbit coupling (SOC), ligand field and surrounding environment (spatial and temporal symmetry). Thus, one can control the MAE and other magnetic properties by tuning these parameters^5^,^37^. Additionally, TM adatoms are found to have large SOC that can give rise to a large MAE. A large magnetic moment and magnetic anisotropy has been observed in Co adsorbed on graphene/Ru(0001) and Co on graphene/Ir(111) surfaces. The induction of magnetism in these system is due to the hybridization of graphene monolayer with the *d*-bands of adatom^38^. Pt-Ir, Os-Ru dimers and adatoms on graphene are found to have even larger MAEs^39^,^40^. However, TM adatoms on graphene tend to aggregate due to very small diffusion energies^41^,^42^. This aggregation can be counterbalanced by the presence of vacancies and extrinsic defects, at the same time inducing magnetism into the system^43^,^44^. Feng *et al.* synthesized reduced graphene oxide (RGO) by chemical exfoliation method and obtained fluorinated RGO by annealing RGO with XeF_2_. The fluorinated graphene is found to be stable and magnetic in the presence of vacancies^45^. The magnetism in such a system can be measured by using inelastic electron tunneling spectroscopy, where the magnetic moment, its orientation and MAE can be measured^37^,^46^.

As defects and doping play an important role in functionalizing graphene^47^, an intensive computational and experimental research has focused on designing new graphene based materials with fascinating physicochemical properties. In recent years, successful synthesis efforts have already been made for B, N and F doped graphene as well as other functional groups doped graphene, which are suitable for numerous technological applications^48^,^49^,^50^,^51^,^52^,^53^,^54^. Recent advances in first-principles based computational methods and high performance computing have given rise to efficient and powerful tools for *in silico* identification of promising defects/impurities with desired properties, and thereby investigating structure-property relationship.

In this work, we investigate the magnetic properties of W atom adsorbed on tri-vacancy fluorinated (TVF) graphene; a schematic picture is shown in Fig. 1(a). As shown in the figure, replacing one graphene ring by three electronegative fluorine atoms at alternate positions polarizes the system through a small charge transfer from W to neighboring C and F atoms. This leads to an overall anisotropic configuration, which results in a large magnetic moment and MAE in the system. Experimentally, such defects can be efficiently realized using a two-step process: adatom is deposited on graphene after creating desired number of vacancies using ion bombardment, pulsed laser or chemical vapor deposition^55^,^56^,^57^. Our study reveals that there is: (i) an induced magnetism in thermodynamically stable W adsorbed TVF graphene, (ii) spin crossover from high-spin to low-spin state under the application of an electric field and thereby the magnetization of the system can be tuned with an electric field, and (iii) a complex interplay between the defect states and chemisorbed metal atom that results in a large magnetic moment and a high in-plane MAE. These findings are of immense importance for designing data storage, non-volatile memory and spintronic devices. We also investigate the quantum mechanical origin of the change in the magnetic properties of W adsorbed TVF graphene with electric field.

## Results and Discussion

### Thermodynamic Stability

Prior to investigate the physicochemical properties of W chemisorbed TVF graphene, we first investigate the thermodynamic stability of TVF graphene. Although nanoporous graphene^2^ and fluorine doped graphene^51^ have been successfully synthesized in experiments, there hasn’t been any theoretical study on the stability of fluorine doped trivacancy graphene. A necessary step for the atomic scale understanding of fluorine incorporation in a nanoporous graphene is the computation of energetics associated with the increasing fluorine content. In order to do that we calculate the defect formation energy (DFE) of fluorine doped graphene as,


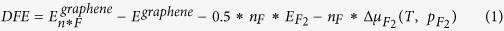


In Eqn. (1), 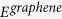 and 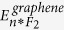 are the DFT total energies of considered pure graphene with six carbon vacancies and fluorine doped graphene sheets; 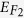 is the DFT energy of an isolated *F*_2_ molecule and *n*_*F*_ is the number of F atoms incorporated. The last term in Eqn. (1) describes the temperature (*T*) and partial pressure 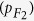 dependence of the chemical potential of fluorine molecule, which includes contributions from the translational, rotational and vibrational degrees of freedom of F_2_, and can be determined via statistical thermodynamics or from thermochemical JANAF Tables^58^.

Using *ab-initio* computations for fluorine doped graphene, the phase diagram showing the defect formation energetics as a function of F_2_ chemical potential (Δμ(F_2_)) is shown in Fig. 2(a). From the phase diagram, it can be seen that the doping of the nanoporous graphene with three F atoms is favored on a wide range of *T*-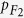 conditions. Figure 2(a) also suggests that Δμ(F_2_) for thermodynamically stable TVF graphene with three fluorine atoms lies between −2.45 to −1.85. In the inset of Fig. 2(a), DFE of nanoporous graphene is plotted as a function of the number of F atoms at the upper and lower bounds of Δμ(F_2_) of thermodynamically stable TVF graphene with three F atoms. To ensure the surface thermodynamic stability of the system under real-world synthesis conditions, (*i.e.* thermal annealing), the dependence of F_2_ chemical potential (Δμ(F_2_)) in the gas phase is translated to the pressure scale at 1100 K.

As depicted in Fig. 2, nanoporous graphene with three F atoms is the most stable configuration. Therefore, we consider TVF graphene, which creates a three-fold coordinated site for the adatom to be adsorbed. This can lead to profound changes in the magnetic properties of the system. We also checked the stability of W adsorbed TVF graphene as compared with other possible configurations. For example, we find that W adsorbed TVF graphene is 0.9 eV lower in energy than a WF_3_ molecule in vacuum and a nanoporous of six C vacancies in graphene. Thus, our calculations, as shown in the Fig. 2(a), confirm the feasibility of these defect formations and suggest experimental follow-up. Furthermore, the binding energy of W on TVF graphene is found to be −2.78 eV, which is calculated as,


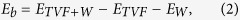


where 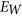 is the total energy of W atom, while 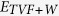 and *E*_*TVF*_ are the total energies of TVF graphene with and without adsorbed W atom, respectively.

In order to investigate the total energy and spin moment of the system at a finite temperature, we performed *ab-initio* MD simulations. In Fig. 2(b), we plot the total energy and spin magnetic moment of the system as a function of time at *T* = 290 K. We find an almost constant energy and a stable ferromagnetic state over the time at *T* = 290 K, which confirms that the system (W adsorbed TVF graphene) is dynamically stable. We also tested the ferromagnetic stability at room temperature (*T* = 300 K) for a few hundred steps and found the system to be stable at room temperature and showing FM properties.

### Magnetic Moment

**Figure 1:**
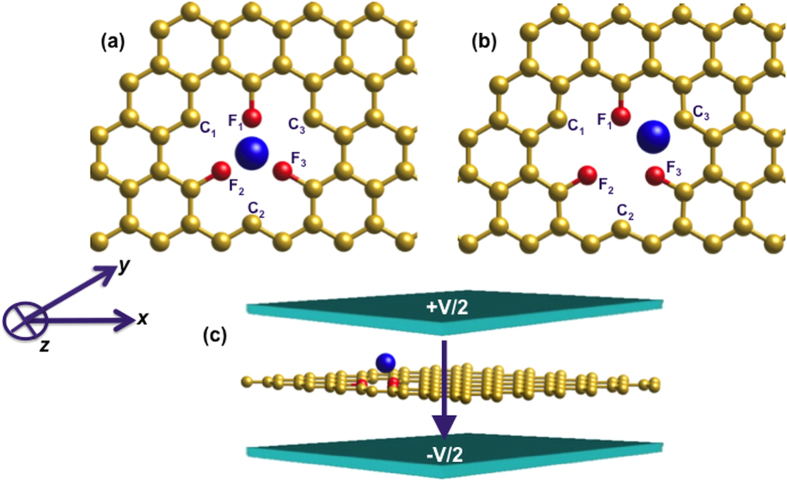
Top view of (**a**) an initial configuration, and (**b**) the relaxed structure of W chemisorbed TVF graphene; alternate carbon atoms are replaced by fluorine; (**c**) W adsorbed TVF graphene layer is placed in an external electric field. The yellow, red and blue spheres correspond to C, F and W atoms, respectively.

**Figure 2:**
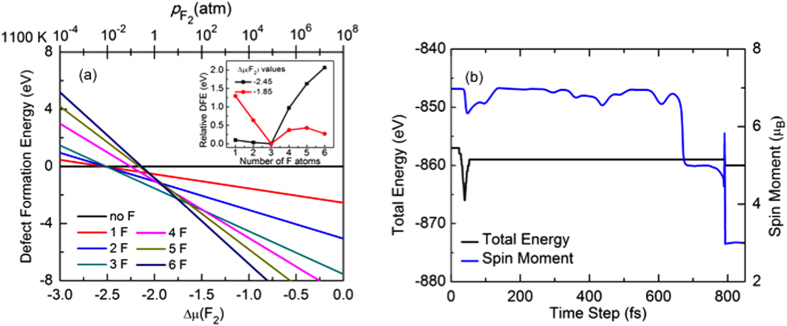
(**a**) Defect formation energetics as a function of fluorine chemical potential (Δμ(F_2_)) for doping of F in graphene. The inset shows the defect formation energies of nanoporous graphene as a function of number of F atoms in the ring. (**b**) Total energy and total spin magnetic moment as a function of number of time steps at *T* = 290 K.

As shown in Fig. 1(a), the dangling bonds unoccupied by F atoms in nanoporous graphene are denoted as C_1_, C_2_, and C_3_. A stable structure after the relaxation process is achieved as a consequence of the repulsion between F atoms and un-bonded carbon atoms that results in W atom getting bonded with one of the un-bnded carbon atoms and breaking the symmetry of the system. This results in lowering the energy of the system and a Jahn-Teller distortion in the system. The geometrical reconstruction of dangling bonds results in symmetry breaking that results in the total spin magnetic moment of the system to be 7 μ_B_. The physical origin of high spin magnetic moment can be inferred from the high-spin configuration of 5*d*-series W (*d*^5↑^, *d*^0↓^, *s*^1↑^, s^0↓^) adatom on TVF graphene.

**Figure 3:**
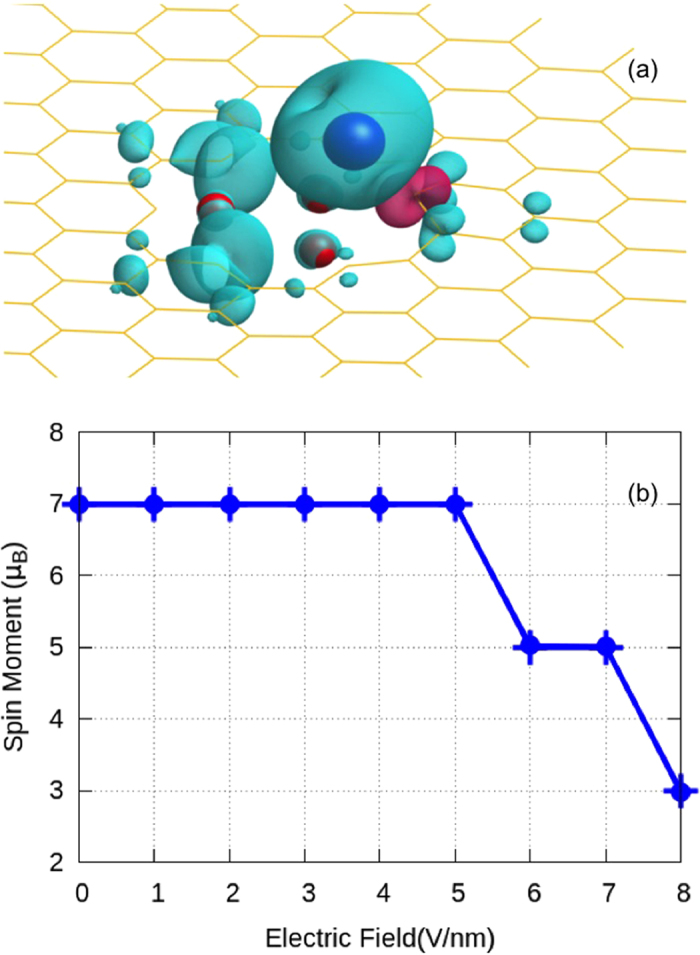
(**a**) Spin density profile of W adsorbed TVF graphene, (**b**) Variation of the total spin moment of the system as a function of electric field, without including spin-orbit coupling in the system.

The valence states of 5*d*-TM atoms have filled 6*s* orbitals and partially filled, half filled or completely filled *d*-orbitals. The electronic configuration of W atom is 5*d*^5↑^, 6*s*^1↑^. Adsorption of W atom on TVF graphene leads to a rise in the energy of 6*s* orbitals, which is then minimized through the polarization of 6*s* orbitals by transferring the charge from 6*s* to the minority states of 5*d*-orbitals^59^,^60^. The covalent bonding between W atom and TVF graphene results into charge transfer from the W to carbon and fluorine atoms as evident from the spin density profile shown in Fig. 3(a); this in turn decreases the magnetic moment of W. From the figure we also find that the W adatom is bonded to one of the carbon atoms (C_3_). The other two dangling bonds remain unsaturated and induce a finite magnetic moment of 0.70 μ_B_/dangling bond which is same as the magnetic moment of the dangling bonds in absence of the adatom. As W is bonded to one of the dangling bonds by hybridization only, most of the uncompensated charges need to be accommodated on W atom itself, which leads to a large moment of 3.70 μ_B_ per W atom. Note that this spin magnetic moment is greater than the magnetic moment of 2.29 μ_B_ previously found for W adatom on di-vacancy nitrogenized graphene^40^.

To check the stability of the hybrid system at high electric fields, we calculate its cohesive energy in the absence and presence of e-field. The calculated cohesive energy per atom of W adsorbed TVF graphene at zero and 6V/nm e-fields are 7.66 and 7.67 eV, respectively. These cohesive energies confirm that the defect can withstand high electric fields. The change in spin magnetic moment of W adsorbed TVF graphene as a function of electric field is shown in Fig. 3(b). Magnetic moment of the system remains constant in the range of 0–5 V/nm e-fields. In this range, both the neighboring dangling bonds are ferromagnetically coupled. However, beyond 5 V/nm, the spin of C_2_ couples antiferromagnetically with C_1_ in the neighboring ring due to change in the occupation of states of both the dangling bonds. This results in decrease in the magnetic moment of the system. Thus the magnetic moment of the hybrid system jumps from a high-spin value to a low-spin value as the electric field changes from 5 to 6 V/nm resulting in a spin crossover effect. As the high- and low-spin states describe the different magnetic states, switching between the two states with an e-field can be used to increase the efficiency and sensitivity of magnetic memory devices.

Quantum mechanically, magnetism of dangling bonds arises primarily from the un-bonded *sp*^2^ orbitals, however a small fraction of this moment also comes from the spin polarized π-orbitals. The electric field shifts the lower edge of the minority states towards the Fermi level, which leads to a decreased magnetic moment contribution by dangling bonds and therefore reduction in the total moment of the system as can be seen in Fig. 3(b). At and beyond an electric field of 8 V/nm, spins of both the dangling bonds couple antiferromagnetically with the W atom and there is also a decrease in the spin moment of W atom due to a change in the occupation of *d*-orbitals. This further decreases the total spin moment of the system. Thus, through electric field, one can control the alignment of spins on carbon atoms in the defect region. Although the electric field affects the magnetic moment of W, the effect is greater on dangling bonds where the coupling of dangling bonds changes from the ferromagnetic to antiferromagnetic.

### Magnetic Anisotropy

**Figure 4:**
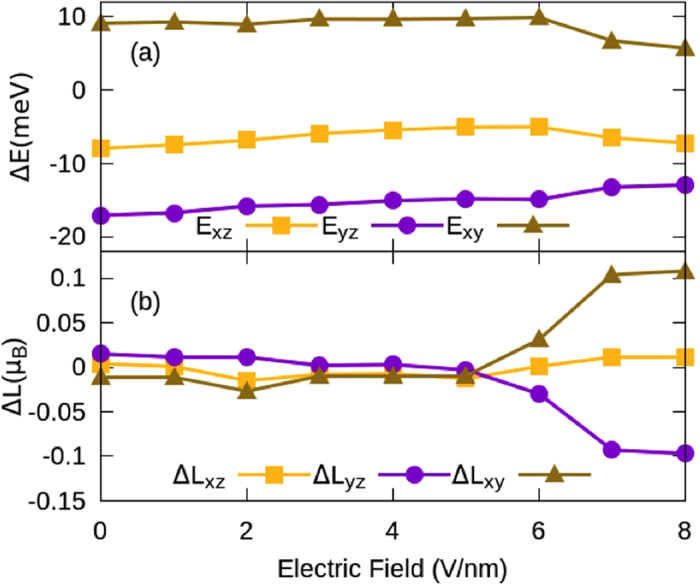
Effect of electric field on the (**a**) energetics and (**b**) orbital moment along three principal magnetization directions in W adsorbed TVF graphene. E_ij_ and ΔL_ij_ are defined in the text.

Despite the large magnetism, the use of any given material in spintronic devices also depends upon its MAE as it decreases the thermal fluctuations in the magnetization of the system. The estimated energy differences for W adsorbed TVF graphene between different magnetization directions are: ΔE_*xz*_(E_*x*_–E_*z*_) = −7.95, ΔE_*yz*_(E_*y*_–E_*z*_) = −17.19 and ΔE_*xy*_(E_*x*_–E_*y*_) = 9.25 meV. This suggests that the easy axis of magnetization lies in the plane of the graphene (along *y*-axis) and hard axis is perpendicular to the surface lying along the *z*-axis with an MAE of 17.19 meV. Energy differences between principal magnetization directions are plotted in Fig. 4(a). We find that although MAE depends upon the occupations of states of the system, here electric field has no significant effect on the MAE (E_*y*_–E_*z*_) until the applied e-field reaches 6 V/nm. Beyond the e-field of 6 V/nm, a gradual variation in MAE has been observed as shown in Fig. 4(a). At an e-field of 8 V/nm, the corresponding energy differences are: E_*x*_–E_*z*_ = −7.20, E_*y*_–E_*z*_ = −12.93 and E_*x*_–E_*y*_ = 5.72 meV. Hence, despite the decrease in the energy differences, the easy and hard directions of magnetizations remain unchanged.

Change in the occupation of *d*-orbitals and change in the orbital moment can affect the values of MAE^5^,^61^. The change in orbital moment with respect to orientation of the magnetization vector from one direction to other namely ΔL_*xz*_ (L_*x*_–L_*z*_), ΔL_*yz*_ (L_*y*_–L_*z*_) and ΔL_*xy*_ (L_*x*_–L_*y*_), in presence of SOC is plotted in Fig. 4(b). Note that ΔL_*yz*_ (L_*y*_–L_*z*_) is negative beyond an e-field of 5 V/nm and increases gradually indicating an increase in the orbital moment along the *z*-direction, which would suggest an easy direction of magnetization to be along *z*-direction. However, since this difference is small (<0.1 μ_B_), it is difficult to predict the direction of easy axis based only upon the difference in orbital moment^62^. The discrepancy can be inferred from the fact that the energy induced by SOC matrices below and above the Fermi level is no longer proportional to the projection of magnetization vector on orbital angular momentum. However, beyond 5 V/nm, a positive and gradual increase in ΔL_*xy*_ (L_*x*_–L_*y*_) and ΔL_*xz*_ (L_*x*_–L_*z*_) suggests a switching of MAE at higher electric field. Inclusion of SOC suppresses the magnitude of spin moment, however it has the same trend as the non-SOC case shown in Fig. 3(b). This is due to the directional dependence of the magnetization vector.

**Figure 5:**
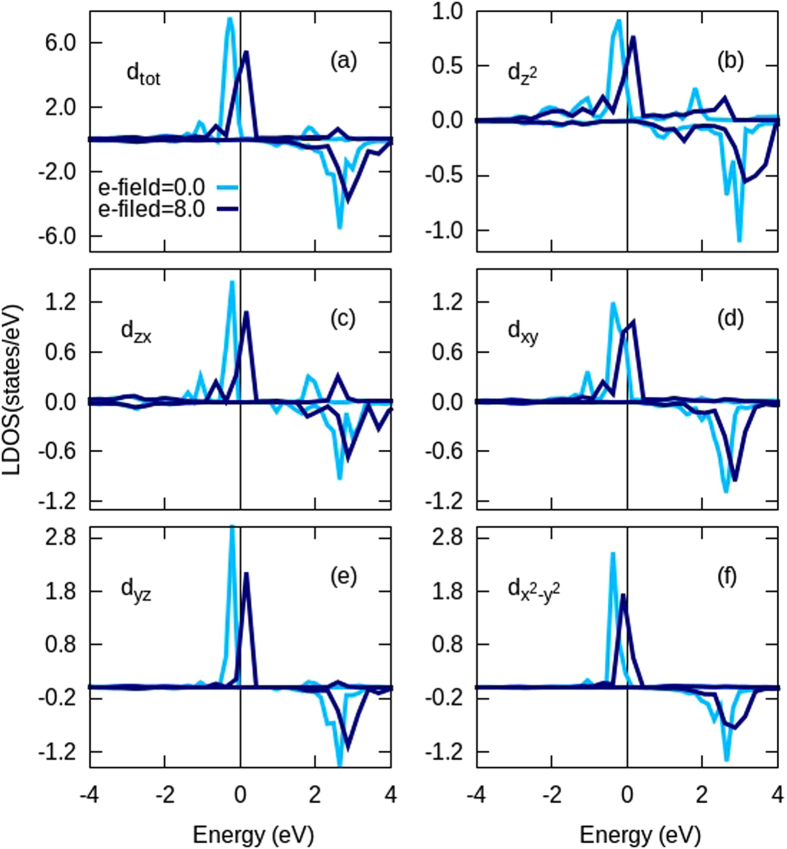
Orbital projected spin-polarized density of states of W adatom on TVF graphene with and without external electric field. The electric field is in V/nm.

To obtain a quantum mechanical picture of the origin of magnetism and MAE, in Fig. 5 we plot the total and partial orbital density of states (PDOS) of W adatom in Fig. 5. We see that electric field pushes the majority states towards Fermi level, which changes the occupation of states resulting in a change in magnetic moment and MAE. The MAE in a physical system arises due to (i) the splitting of degenerate partially occupied eigenstates connected through SOC and (ii) by coupling of the states above and below the Fermi level (unoccupied and occupied) through SOC matrix elements^5^. Quantitatively, within second order perturbation, MAE can be expressed as:


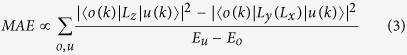


where 〈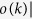 and 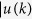
〉 are the occupied and unoccupied eigenstates, respectively; *L*_*z*_ and *L*_*y*_ (*L*_*x*_) are the angular moment operators along the principal axes. As, Eqn. 3 contains energy difference between the unoccupied and occupied states in the denominator; states near the Fermi level will result in a large contribution to Eqn. 3 and the MAE will increase.

The DOS in Fig. 5 also reveal that in the absence of e-field the coupling between *d*_*zx*_ and *d*_*x*_^*2*^_*–y*_^*2*^ and the coupling between *d*_*z*_^*2*^ and *d*_*xz*_ through *L*_*y*_ have dominant contribution to Eqn. (3), which forces the easy axis to be in-plane along *y*-axis. Although application of electric field increases the coupling between (*d*_*zx*_, *d*_*x*_^*2*^_*–y*_^*2*^) and (*d*_*z*_^*2*^, *d*_*xz*_) orbtials, we find a decrease in the MAE of the system at higher fields. This decrease is found to be due to the increase in the coupling interaction of (*d*_*xy*_, *d*_*x*_^*2*^_*–y*_^*2*^) and (*d*_*xz*_*, d*_*yz*_) orbitals through *L*_*z*_^61^. At higher fields, change in the direction of MAE from *y-* to *x*-axis is expected to be due to the coupling of *d*_*xy*_ with *d*_*xz*_, coupling of *d*_*x*_^*2*^_*–y*_^*2*^ with *d*_*yz*_ and the coupling of *d*_*z*_^*2*^ with *d*_*yz*_ orbitals realized through *L*_*x*_ operator.

To check the robustness of presented results with respect to the change in exchange correlation potential, we also performed DFT calculations using PBE-sol pseudopotentials. We find that both PBE and PBE-sol yield similar results for structure and magnetism in the absence of SOC at all the fields. Upon inclusion of SOC, the computational cost of calculations increases, therefore we calculated the MAE and orbital moments just at the smaller fields and found that the results change by 2–3 meV, however the overall trend remains the same.

## Conclusions

In summary, we have investigated the magnetic properties of tungsten adsorbed on nanoporous TVF graphene and have demonstrated the influence of intrinsic defects on the stabilization of the adatom and inhibition of aggregation. Our computational results suggest that the proposed defect is thermo-dynamically and geometrically stable and can be fabricated with existing experimental techniques. Further, we predict that the magnetic properties of the hybrid system can be modified using e-field via the changes in the occupation of W adatom states. As a result of this modulation, we find a switching between high-spin and low-spin states after an e-field of 5 V/nm. Present study also confirms an easy plane anisotropy accompanied by spin crossover in tungsten adsorbed on TVF graphene, which is an important result for non-volatile memory and spintronic devices. Further, we expect to see a change in the direction of magnetization at higher fields or by substituting different adatoms. We believe that our results will stimulate experimental work on these kinds of hybrid systems to develop highly efficient data storage and spintronic devices based on graphene.

### Computational Details

Density functional theory calculations are performed using frozen core projector augmented wave method as implemented in Vienna *Ab-Initio* Simulation Package (VASP)^63^,^64^. Exchange and correlation interactions are modeled using generalized gradient approximation with the Perdew-Burke-Ernzerhof (PBE) functional^65^. To expand the crystal wave functions, a plane wave basis cutoff of 520 eV is used. To accurately estimate the MAE, SOC is used in a noncollinear mode^66^. An energy convergence threshold of 10^−7^ eV and a Γ centered *k*-mesh of size 7 × 7 × 1 is used to sample the Brillouin zone of the system^67^. The geometrical structure is constructed using 7 × 7 × 1 sized supercell.

As demonstrated in the Fig. 1(a), to create TVF graphene a ring containing six carbon atoms is removed and three strong ligand atoms like fluorine are doped at alternate positions. W atom is placed at the center of the ring having ^3^D_h_ symmetry. The other three C atoms in the ring remain non-bonded and are called as dangling bonds. The relaxation breaks the ^3^D_h_ symmetry of the system and the W atom shifts towards one of the carbon atoms, as depicted in Fig. 1(b). The structural relaxation is performed until all the forces on atoms are smaller than 0.001 eV/Å. To study the electric field response of the system, we used dipole layer method^68^ as shown in Fig. 1(c).

To check the stability of W adsorbed TVF graphene system, we also performed finite temperature *ab-initio* molecular dynamic (MD) simulations using Nose-Hoover heat bath scheme^69^ at 290 K and 300 K to check the stability of the system near and at room temperature for 850 steps. The time step between consecutive steps was set to be 1fs.

## Additional Information

**How to cite this article**: Kumar, P. *et al.* Tunable magnetism in metal adsorbed fluorinated nanoporous graphene. *Sci. Rep.* **6**, 31841; doi: 10.1038/srep31841 (2016).
